# Long-Term Outcomes and Risk Factors of Renal Failure Requiring Dialysis after Heart Transplantation: A Nationwide Cohort Study

**DOI:** 10.3390/jcm9082455

**Published:** 2020-07-31

**Authors:** Tsai-Jung Wang, Ching-Heng Lin, Hao-Ji Wei, Ming-Ju Wu

**Affiliations:** 1 School of Public Health, The University of Texas Health Science Center (UTHealth), Houston, TX 77030, USA; s18901005@gmail.com; 2 Department of Medical Research, Taichung Veterans General Hospital, Taichung 407, Taiwan; epid@ms39.hinet.net; 3 Department of Cardiovascular Surgery, Cardiovascular Center, Taichung Veterans General Hospital, Taichung 407, Taiwan; weihaoji@vghtc.gov.tw; 4 Division of Nephrology, Department of Internal Medicine, Taichung Veterans General Hospital, Taichung 407, Taiwan; 5 School of Medicine, Chung Shan Medical University, Taichung 402, Taiwan; 6 Rong Hsing Research Center for Translational Medicine, Institute of Biomedical Science, College of Life Science, National Chung Hsing University, Taichung 402, Taiwan; 7 Graduate Institute of Clinical Medical Science, School of Medicine, China Medical University, Taichung 404, Taiwan

**Keywords:** acute kidney injury (AKI), chronic kidney disease (CKD), dialysis, heart transplantation (HT), immunosuppressant, mortality, renal failure, risk factor, survival analysis

## Abstract

Acute kidney injury and renal failure are common after heart transplantation. We retrospectively reviewed a national cohort and identified 1129 heart transplant patients. Patients receiving renal replacement therapy after heart transplantation were grouped into the dialysis cohort. The long-term survival and risk factors of dialysis were investigated. Patients who had undergone dialysis were stratified to early or late dialysis for subgroup analysis. The mean follow-up was five years, the incidence of dialysis was 28.4% (21% early dialysis and 7.4% late dialysis). The dialysis cohort had higher overall mortality compared with the non-dialysis cohort. The hazard ratios of mortality in patients with dialysis were 3.44 (95% confidence interval (CI), 2.73–4.33) for all dialysis patients, 3.58 (95% CI, 2.74–4.67) for early dialysis patients, and 3.27 (95% CI, 2.44–4.36; all *p* < 0.001) for late dialysis patients. Patients with diabetes mellitus, chronic kidney disease, acute kidney injury, and coronary artery disease were at higher risk of renal failure requiring dialysis. Cardiomyopathy, hepatitis B virus infection, and hyperlipidemia treated with statins were associated with a lower risk of renal dysfunction requiring early dialysis. The use of Sirolimus and Mycophenolate mofetil was associated with a lower incidence of late dialysis. Renal dysfunction requiring dialysis after heart transplantation is common in Taiwan. Early and late dialysis were both associated with an increased risk of mortality in heart transplant recipients.

## 1. Introduction

Heart transplantation (HT) remains the gold standard treatment of advanced heart failure. The prognosis of HT has steadily improved with a recent median survival of 12.5 years among adult HT recipients [1]. A study reported an 82% one-year survival rate and a 69% five-year survival rate after HT [2]. Kidney disease after HT has become increasingly prevalent as outcomes following HT have improved [3]. Renal dysfunction in HT has a broad spectrum, including chronic kidney disease (CKD), end-stage renal disease (ESRD), and acute kidney injury (AKI). Schwarz et al. [4] determined that the majority of biopsy-diagnosed renal disease after transplantation of other organs and tissues was related to hypertensive nephrosclerosis and chronic calcineurin inhibitor (CNI) toxicity. The cumulative incidence of ESRD after HT is from 3.1% to 11% [2,5,6,7,8]. Extensive literature indicates that CKD [9,10] and ESRD [5,11] are independent risk factors for mortality in HT recipients. 

However, evidence has indicated that HT may also cause AKI, and factors causing AKI and CKD may differ. AKI is frequently encountered in HT with the reported incidence being up to 76% [12]. depending on the definition. The incidence of the most severe form, AKI requiring dialysis (AKI-D), ranges from 4% to 28% [13,14,15,16,17]. Patients who require postoperative dialysis exhibit higher rates of postoperative complications, short-term in-hospital mortality [18], and subsequent CKD [8]. Contrary to CKD and ESRD, few studies had evaluated the long-term prognostic implications of the development of AKI and AKI-D in HT recipients. 

Presently, the national cohort of Taiwan is the largest heart HT database in Asia [19]. Therefore, we conducted a retrospective study on the long-term consequences and risk factors of post-HT renal failure requiring dialysis, using the nationwide cohort. The aims of this study were as follows: (1) determine the incidence of dialysis-requiring renal failure after HT; (2) assess if post-HT renal failure requiring dialysis is associated with a higher risk of long-term mortality; (3) characterize the risk factors related to renal failure requiring dialysis after HT; (4) examine crucial determinants associated with the need for early- and late-stage dialysis after HT.

## 2. Experimental Section

### 2.1. Sources of Data

The database used in this study was obtained from the National Health Insurance Research Database (NHIRD). The NHIRD is maintained by the Department of Health and the National Health Research Institutes of Taiwan and consists of ambulatory care records, inpatient care records, catastrophic illness registration, and the registration files of insured patients. Over 99% of the Taiwanese population (23-million beneficiaries) was covered by National Health Insurance (NHI) during the study period [20]. Diagnostic and procedure codes follow the International Classification of Disease, Revision 9, Clinical Modification (ICD-9-CM). We conducted a retrospective observatory study in HT recipients of Taiwan with a focus on renal failure requiring dialysis. This study was approved by the Institutional Review Board of Taichung Veterans General Hospital (CE13151B-4). The informed consent was waived because the data were anonymous.

### 2.2. Study Subjects and Study Design

**Figure 1:**
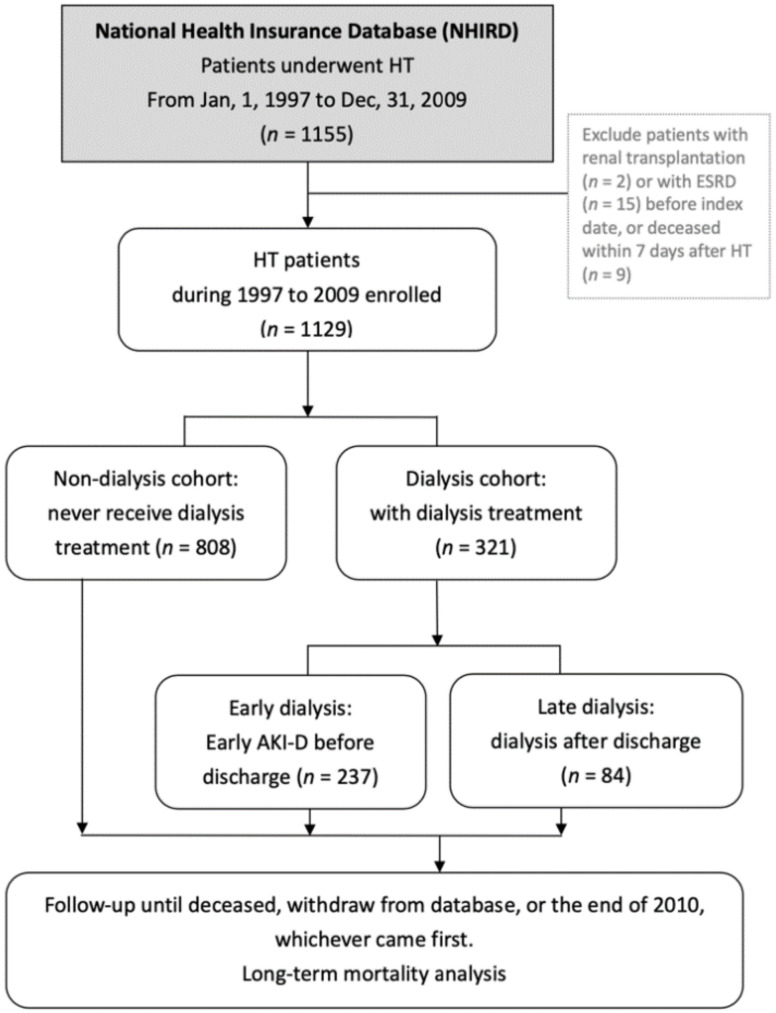
Flow chart of patient selection. Abbreviations: HT, heart transplantation; ESRD, end-stage renal disease; AKI-D, acute kidney injury requiring dialysis.

NHIRD data were extracted for all patients who underwent HT in Taiwan in 1997–2009. A total of 1155 patients were identified. We excluded 2 patients with preceding renal transplantation, 15 patients with ESRD before HT, and 9 patients who died within 7 days after HT, modified and in accordance with previous studies [12]. Therefore, 1129 patients were included in statistical analyses (Figure 1). Recipients were characterized by sex, age, pre-HT comorbidities, primary diagnosis for HT, and immunosuppressant usage. The ICD-9-CM codes for comorbidities and primary diagnosis for HT included hepatitis B (HBV, 070.2–070.3), hepatitis C (HCV, 070.44, 070.51, 070.54, and 070.7), cirrhosis (571), diabetes mellitus (DM, 250), CKD (585), hypertension (HTN, 401–405), hyperlipidemia (272), AKI (584), congenital heart disease (CHD, 745–747), cardiomyopathy (425), and coronary artery disease (CAD, 410–414, 428, and 429.7). We categorized the primary diagnosis for HT to CHD, CAD, and cardiomyopathy for risk stratification for renal failure requiring dialysis. If recipients did not have the aforementioned diagnosis, they were designated to the fourth category as “other”. Subjects were defined as having immunosuppressant exposure if they received prescriptions during the inpatient hospitalization period when HT performed, or at the outpatient service in the year post-HT. 

The primary outcome was renal failure requiring dialysis. Patients were grouped into the dialysis cohort if they had required renal replacement therapy (RRT) after HT. Otherwise, they were categorized into the “non-dialysis” cohort. The secondary outcome was mortality throughout the follow-up period. The endpoint of follow-up was the ending date of NHI coverage or the end of the study on 31 December 2010. The ending date of NHI coverage was demonstrated to be a suitable proxy for patient survival [21].

### 2.3. Subgroup Definitions

The dialysis cohort was further stratified by the time at which dialysis was performed. “Early” dialysis was defined as renal failure mandating dialysis during the immediate post-transplantation period and before discharge. Dialysis performed during hospitalizations after discharge from the index HT admission was defined as “late” dialysis. The stratified groups were used for subgroup analysis to investigate survival differences and risk factors.

### 2.4. Statistical Analysis

Data are expressed as mean ± standard deviation unless otherwise specified. Student’s t-tests were used to investigate differences in continuous variables between the dialysis and non-dialysis cohorts. Categorical parameters were expressed as number and percentage and compared using Chi-square tests. Follow-up time (in person-years) was calculated for each subject from the initial HT until death or the end of the study. The incidence rates of mortality were calculated in the follow-up period until the end of 2010. Survival data were analyzed and compared using the Kaplan–Meier method and the log-rank test, respectively. An overall survival curve and survival curve conditional on survival to 3 months after HT were constructed for each group. The multivariate Cox’s proportional hazard regression was used to examine the effect of dialysis exposure on the risk of mortality, presented as hazard ratios (HRs) with 95% confidence intervals (CIs). The multivariate Cox’s proportional hazard regression was also used to explore risk factors for renal failure requiring dialysis. A two-tailed *p*-value of <0.05 was considered statistically significant. All statistical analyses were performed using SAS statistical software (version 9.2 for Windows; SAS Institute, Inc., Cary, NC, USA). 

## 3. Results

### 3.1. Incidence of Renal Failure Requiring Dialysis after HT

Table 1 presents the baseline characteristics of the 1129 HT recipients and dialysis data. The mean age was 45.5 ± 16.8 years. The leading etiology of HT was unspecified cardiomyopathy (40%), followed by CAD (24.6%). The mean follow-up duration was 5.0 ± 4.1 years. Among the HT recipients, 808 (71.6%) had never received dialysis (non-dialysis group). Renal failure requiring dialysis had developed in the 321 (28.4%) patients (dialysis group). The incidence of renal failure requiring dialysis was 3.36 per 100 patient-years among HT recipients. A larger proportion of HT recipients who received dialysis were men compared with the non-dialysis cohorts (79.4% vs. 66.5%, *p* < 0.001). Significant differences were also observed in HBV carrier status, cirrhosis, DM, CKD, HTN, primary diagnosis for HT, and immunosuppressant usage between the dialysis and non-dialysis cohorts (*p* < 0.05).

### 3.2. Mortality in Patients with or without Need for Dialysis after HT

**Figure 2:**
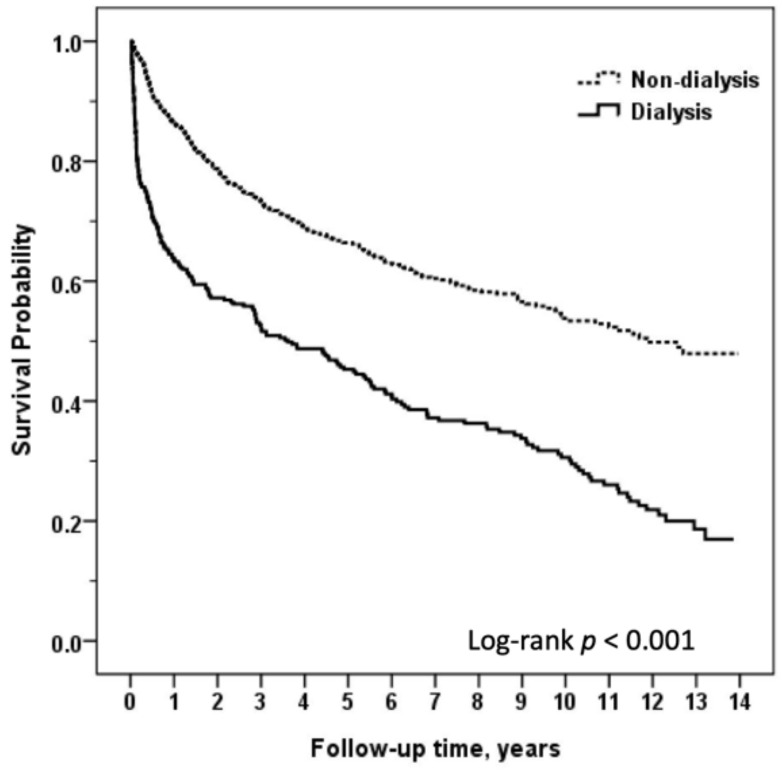
Comparison of long-term survival between heart transplant recipients with or without dialysis.

Overall patient survival was 79.9% after one year, 68.1% after three years, and 62.7% after five years. The mortality outcomes for HT patients by dialysis status are summarized in Table 2. Sex, age, and significant variables in Table 1 were included in the logistic regression model. The dialysis group exhibited higher overall mortality compared with the non-dialysis group (HR: 3.44, 95% CI: 2.73–4.33). The one-year (HR: 5.89, 95% CI: 4.09–8.48), three-year (HR: 4.03, 95% CI: 3.03–5.34), and five-year (HR: 3.68, 95% CI: 2.83–4.78) mortality from any cause by Kaplan–Meier analysis were all significantly higher in the dialysis cohort than in the non-dialysis cohort (Table 2 and Figure 2). This increase in mortality was most pronounced in the first year post-HT and decreased considerably over time, up to five years post-HT.

### 3.3. Timing of Dialysis Delivery and the Consequences after HT

The dialysis group (*n* = 321) was divided into two subgroups based on when dialysis was delivered; 237 (21% of all HT recipients) patients required early dialysis and 84 (7.4% of the entire study population) patients required late dialysis. The mean duration from HT to late dialysis was 3.4 ± 3.2 years. Maintenance RRT was performed in 52 patients (30 in the early group and 22 in the late group). Patients with incident ESRD accounted for 16.2% of the dialysis cohort and 4.6% of the entire study population. The mean duration from HT to ESRD was 6.0 ± 3.8 years. The overall mortality in both early (HR: 3.58, 95% CI: 2.74–4.67) and late dialysis (HR: 3.27, 95% CI: 2.44–4.36) was significantly higher than the mortality of the non-dialysis cohort. However, the mortality difference between the early and late groups was not significant (*p* = 0.220). Data are presented in Table 3. 

**Figure 3:**
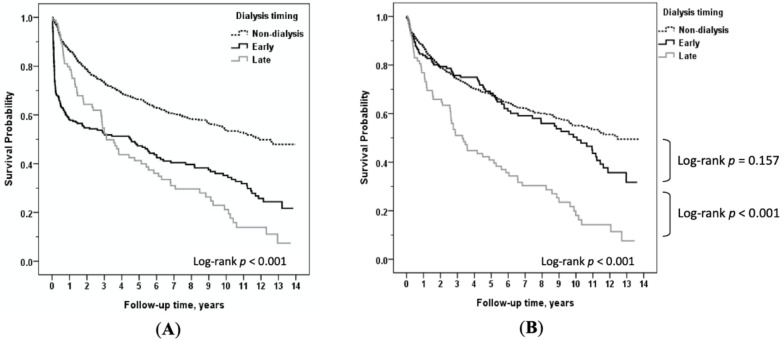
(**A**) Comparison of long-term survival among heart transplant recipients with non-dialysis versus early dialysis versus late dialysis; (**B**) among non-dialysis versus early dialysis versus late dialysis, conditional on survival to 3 months after heart transplantation.

The Kaplan–Meier survival curve plots of the time course of deaths after HT in patients with early dialysis, late dialysis, or without dialysis are illustrated in Figure 3. As displayed in Figure 3A, differences in survival were most pronounced during the first three months after surgery (*p* < 0.05), especially among patients who developed early severe AKI-D. We generated Figure 3B to illustrate the survival differences among the three-month survivors in different subgroups. Conditional survival analyses revealed that survival did not differ between the early dialysis and the non-dialysis groups (*p* = 0.157) if they survived at least three months. However, the mortality of the late dialysis cohort remained relatively high throughout the entire observation period.

### 3.4. Risk Factors of Renal Failure Requiring Dialysis

The multivariable Cox regression analysis revealed that the baseline characteristics and preoperative predictors for renal failure requiring dialysis among HT patients were HBV infection (OR: 0.32, 95% CI: 0.14–0.76), pretransplantation DM (OR: 1.5, 95% CI: 1.07–2.11), preexisting CKD (OR: 2.65, 95% CI: 1.46–4.79), history of AKI (OR: 2.36, 95% CI: 1.27–4.41), and primary HT diagnosis of CAD (OR: 10.07, 95% CI: 6.33–18.4) (Table 4). 

We further examined the risk factors of early and late dialysis, respectively. The statistically significant comorbid risk factors of AKI requiring early dialysis were CKD (OR: 2.84, 95% CI: 1.54–5.24), AKI (OR: 3.00, 95% CI: 1.56–5.78), and primary HT diagnosis of CAD (OR: 17.29, 95% CI: 9.17–32.6). Factors associated with a lower risk of early dialysis were HBV (OR: 0.37, 95% CI: 0.15–0.93), hyperlipidemia treated with a statin (OR: 0.63, 95% CI: 0.4–0.99), and heart failure caused by cardiomyopathy (OR: 0.56, 95% CI: 0.38–0.83), compared with patients who did not receive dialysis (Table 5). Clinical variables significantly associated with late dialysis were the prescription of sirolimus in contrast to no use (OR: 0.17, 95% CI: 0.08–0.36) and taking mycophenolate mofetil (MMF) instead of azathioprine (OR: 0.26, 95% CI: 0.13–0.51). The immunosuppressants sirolimus and MMF were associated with lower risks of late dialysis (Table 6).

## 4. Discussion

The overall rate of renal failure requiring dialysis throughout the follow-up period (28.4%), the AKI-D during the early perioperative phase (early dialysis, 21.0%), and the cumulative incidence of ESRD (4.6%) were high in our cohort. The incidence of early dialysis and ESRD accorded with previous studies.

Our objective was to analyze the effect of post-HT renal failure requiring dialysis on long-term survival. This cohort is one of the largest to have been used to investigate this subject. We observed a significantly increased risk of mortality in HT recipients on dialysis, compared with HT recipients without dialysis. Early and late dialysis were both independent predictive factors for patient mortality in our subgroup survival analysis. 

Boyle et al. [18] reported that the incidence of postoperative AKI-D in a single center in the USA was 5.8%. The reported in-hospital mortality rate was very high at 50%, which contrasted starkly to the mortality rate of 1.4% in patients who did not develop AKI-D (*p* < 0.001). Unlike robust evidence of the association of AKI-D and poor short-term in-hospital mortality after HT [16,18,22], only three single-center studies have assessed the effect of AKI after HT on long-term survival [17,23,24], and the results of these studies were conflicting. Fortrie et al. reported their findings from 471 HT recipients in the Netherlands [17]. AKI was defined and staged by the Kidney Disease Improving Global Outcome (KDIGO) classification. During the first postoperative week, 75.4% of the recipients developed AKI, and RRT was required in 4% of recipients. AKI-D was associated with an increased risk for mortality (HR = 2.59, 95% CI = 1.17–5.73) but less severe episodes of AKI did not affect the recipient’s long-term prognosis, with a median follow-up of 9.5 years and a conditional survival of one year. A study in Spain revealed that 40.3% of patients among 310 HT recipients developed AKI, as defined using the KDIGO criteria [23]. Overall long-term survival among patients with AKI-D and patients who developed AKI without dialysis requirements was not significantly different (*p* = 0.1). Gude et al. [24] reported a similar nonsignificant long-term survival effect of AKI-D in a cohort from Norway. 

With a maximum 14-year follow-up, our findings suggest the overall poor long-term survival in HT patients with renal failure requiring dialysis and provide new evidence that a substantial percentage of early AKI-D three-month survivors had relatively long-term survival after the acute phase of their illness. Nevertheless, the late dialysis subgroup exhibited a persisted high risk of mortality throughout the study period. This finding was somewhat surprising and may reflect a high proportion of the three-month survivors recovering from early AKI-D. Among the 237 early dialysis patients, 30 (12.7%) recipients died during the index admission, 30 (12.7%) recipients developed ESRD during the follow-up period, and the majority of patients (*n* = 177, 74.7%) recovered sufficient kidney function to discontinue dialysis. Compared with the late dialysis cohort (ESRD in 26% of patients), recipients in need of early dialysis had a lower risk of the ongoing need for dialysis after the acute stage (*p* = 0.004, Chi-square test). Recovery of cardiac function in HT survivors may mitigate the severe perioperative renal insults encountered. These findings indicate that measures to protect and restore kidney function during and after an episode of AKI-D are greatly needed. 

The multivariable analysis identified pretransplantation DM, CKD, history of AKI, and end-stage heart failure caused by CAD as independent predictors of the need for dialysis. Moreover, we observed that the determinants of early and late dialysis differed. We observed a significant association between HT diagnosis and early dialysis. CAD as the primary diagnosis for HT was a risk factor for early dialysis, whereas patients who underwent HT due to cardiomyopathy were associated with a lower risk of early dialysis. Regardless of the renal function, HT caused by CAD was associated with lower long-term survival than cardiomyopathy in our cohort (Figure S1). However, the relationship between the primary diagnosis of HT and early AKI-D has not been identified yet despite its prognostic values for survival. Martinelli et al. [25] evaluated 128 HT patients with ischemic cardiomyopathy and 147 with idiopathic dilated cardiomyopathy. They reported that patients with ischemic cardiomyopathy had a more critical stay in the hospital, which echoes our findings that up to 222 out of 278 (80%) patients with CAD required early dialysis. 

Notably, hyperlipidemia alone was not a significant indicator for overall dialysis, early dialysis, or late dialysis in our cohort. However, hyperlipidemia treated with statins was independently associated with a reduced risk of early dialysis. The survival benefits of statins among patients undergoing cardiac surgery, not including HT, has been debated [26]. Hydroxy-methylglutaryl-coenzyme A (HMG-CoA) reductase inhibitors (statins) have also been reported to increase the risk of AKI in non-HT cardiac surgery [27]. However, previous researches suggest that patients who received statin therapy before HT had a lower risk of CKD after HT [14,28]. Instead of prescribing routinely to all HT recipients in many countries, the Taiwan National Health Bureau would only reimburse initial statin prescriptions for patients if the total cholesterol was ≥200 mg/dL or the low-density lipoprotein (LDL)-cholesterol was ≥130 mg/dL during the study period. Our study added new evidence of the favorable association of statin use in HT recipients with hyperlipidemia and early postoperative AKI-D. Statins affect several mechanisms underlying post-HT AKI, including attenuation of myocardial ischemia-reperfusion injury, decreased inflammatory markers, and alteration of cyclosporine level [29]. The effect of statin continuation in recipients already using statins or de novo initiation of perioperative statin treatment in recipients naïve to statins on AKI among patients post-HT is unclear. Our observations linking statin use to a lower risk of early AKI-D in HT recipients merits further investigation.

Immunosuppressants are particularly crucial because CNI toxicity has been associated with posttransplant renal failure [30] and may be involved in the development of thrombotic microangiopathy (TMA) [4]. Moreover, the mammalian targets of rapamycin (mTOR) inhibitors are associated with an increased risk of proteinuria, but may be desirable considering its association improved renal function [13]. In the present study, the risk of late dialysis was lower among HT recipients who were treated with sirolimus compared with HT recipients who were not treated with sirolimus, and the risk was lower in patients with MMF over azathioprine. Our results accord with findings by Jokinen et al. [14], who reported that the use of azathioprine instead of MMF was the strongest predictor for impaired renal function at 12 months after HT.

In our cohort, the association with a lower risk of late dialysis in patients with Sirolimus may come from the substitution of CNI. The use of mTOR inhibitors in place of CNI could be considered in patients with renal impairment or progressive CNI toxicity [31,32]. However, data regarding the outcomes in HT patients treated with mTOR inhibitors are controversial and adverse events are often reported. 

The SCHEDULE trial [33], the first randomized multicenter trial to evaluate an early CNI withdrawal in HT recipients, reported adequate immunosuppressive potency and a sustained renal advantage using the Everolimus-based regimen over CNI. A meta-analysis [34] reported that a mTOR/MMF combination preserves renal function but increases the risk of acute cellular rejection. Although the cause of devastating renal failure requiring late dialysis may be multifactorial in origin, it was strongly associated with the use of immunosuppressants in our cohort. When devising strategies to prevent late-onset renal failure requiring dialysis in HT recipients, mTOR/MMF immunosuppressants may be of value. 

CKD often predates transplantation in patients with end-stage organ disease, deteriorates after transplantation, and increases the risk of AKI and dialysis dependency [10]. In our study, pretransplant CKD was associated with an elevated risk of dialysis and early dialysis after transplantation. However, the effects were not evident among patients with late dialysis. A multi-institutional study on late renal dysfunction after pediatric HT reported that renal function at HT was not associated with the onset of late renal dysfunction [35]. Although our findings indicate that the effect of preexisting CKD on postoperative renal dysfunction decreases over time in HT recipients, CKD is considered a useful indicator for assessing the risk of early AKI-D, drug dosing, stratifying comorbidities, and assessing the long-term toxicity of immunosuppression. 

The high prevalence of CKD and low CKD awareness among both physicians and the general population have been documented in Taiwan and worldwide [36,37,38,39]. A study by Chen et al., including 248 HT recipients in a single center in Taiwan, reported renal dysfunction before transplant in 8% of patients [40]. This serum creatinine-based CKD diagnosis rate was not significantly different from our pre-HT CKD prevalence (5.1%) according to a Chi-square test (*p* = 0.07). However, if CKD in Chen’s cohort was defined by eGFR < 60 mL/min/1.73 m^2^, the proportion of pretransplant CKD increased (36%), which suggests that CKD burden may be underestimated in our study and other clinical settings. Early and timely detection of renal dysfunction before and after HT may help to minimize renal insults.

Surprisingly, HBV carriers had a lower risk of dialysis-requiring renal failure and early dialysis after HT. HBV is hyperendemic in Taiwan where organ donors or recipients with positive serum hepatitis B surface antigen are frequently encountered. However, the cause of the relationship between HBV and AKI in HT recipients remains unclear. Ko et al. reported a single-center cohort in Taiwan. They found HBV reactivation after HT is common but usually well controlled with antiviral treatment [41]. Therefore, HBV carrier status should not contraindicate HT. 

Studies on risk factors for AKI during the immediate post-HT period were conducted on single-center cohorts and most were inconclusive. To date, our study investigated the largest cohort regarding this subject. Moreover, we observed differences in risk factors associated with the most severe form of renal failure at both early and late stages after HT in the same cohort. Despite the lack of intraoperative information, we collected comprehensive data on underlying comorbidities. Furthermore, our results provided new information and insights regarding AKI risk identification before HT. To further clarify the risk factors predicting early AKI, we summarized and compared the risk factors predicting early postoperative AKI between our cohort and published studies. The results are summarized in Table S1 [12,14,18,23,24,42].

This study has several limitations. First, we present observational data from a national cohort, which only reveals associations, not causality, which should be considered when interpreting the results. Second, we excluded early postoperative death within seven days, modified, and in accordance with previous studies [12]. Although it allows us to focus on long-term survival analysis, this makes our study a “landmark” of subjects surviving for more than seven days. Third, the variation of HT and dialysis indications between physicians and hospitals may be present in a large multicenter database. In Taiwan, every operation of HT was assessed by the Bureau of NHI preoperatively. Furthermore, a retrospective inspection of dialysis indication and routine ESRD eligibility evaluation may lower the bias caused by inter-center differences. Fourth, data regarding the acuteness or chronicity of renal failure was not available. We considered a severe clinical scenario and applied a single definition of AKI (renal failure requiring dialysis) for HT recipients. We aimed for an accurate representation of early AKI-D cases by excluding patients with an ESRD diagnosis on admission. Early dialysis is widely speculated to be the most severe form of AKI after HT. However, differentiating true AKI from progressive CKD may be a challenge in late dialysis, both in this study and in clinical settings. Despite this limitation, understanding the risks and outcomes of late dialysis permits the institution of proper treatment. Fifth, certain variables of interest were not documented in this database, such as laboratory tests, patient severity, time to transplant, and perioperative conditions. Those who are sicker and had a longer time to transplant will not do as well as those who received it early. We recognize these limitations and the inability to adjust for all potential confounders. Last, the percentage of CNI use was lower than expected in the non-dialysis cohort (Tacrolimus or cyclosporine in 58.5% of the non-dialysis group and 93.5% in the dialysis group). A minority of the remaining recipients may use CNI-free regimens, the majority may be attributed to clinical trials. Patients who did not require dialysis were potential participants in clinical trials on immunosuppressants, offered by a third party, which may have lessened the magnitude of risk estimates of CNI usage.

## 5. Conclusions

In conclusion, renal failure requiring dialysis after HT is common in Taiwan. Although early and late dialysis were both associated with an increased risk for mortality, early postoperative AKI requiring dialysis was not associated with a less favorable long-term outcome if recipients survived beyond three months. Risk factors of early and late dialysis differed. Hyperlipidemia treated with statins and HT caused by idiopathic cardiomyopathy were associated with a lower early postoperative dialysis risk, whereas sirolimus or MMF use was associated with a lower late dialysis risk. The heterogeneity of risk factors suggests different mechanisms of renal failure in the early and late stages after HT. Risk factors recognition aids the development of kidney protective strategies.

## Figures and Tables

**Table 1:** Demographic and clinical characteristics between HT recipients with or without dialysis.

	Total (*n* = 1129)		Nondialysis (*n* = 808)		Dialysis (*n* = 321)		
Variables	*n*	(%)	*n*	(%)	*n*	(%)	*p* ^a^
**Sex, men**	792	(70.2)	537	(66.5)	255	(79.4)	<0.001
**Age, years**							0.088
Mean ± SD	45.5 ± 16.8		45.3 ± 17.0		46.1 ± 16.3		
<18	97	(8.6)	69	(8.5)	28	(8.7)	
18–39	272	(24.1)	211	(26.1)	61	(19.0)	
40–59	539	(47.7)	373	(46.2)	166	(51.7)	
≥60	221	(19.6)	155	(19.2)	66	(20.6)	
**Comorbidities**							
HBV	48	(4.3)	41	(5.1)	7	(2.2)	0.030
HCV	17	(1.5)	13	(1.6)	4	(1.2)	0.652
Cirrhosis	186	(16.5)	122	(15.1)	64	(19.9)	0.048
DM	279	(24.7)	166	(20.5)	113	(35.2)	<0.001
CKD	58	(5.1)	22	(2.7)	36	(11.2)	<0.001
Hypertension	449	(39.8)	296	(36.6)	153	(47.7)	<0.001
Hyperlipidemia	271	(24.0)	184	(22.8)	87	(27.1)	0.124
AKI	52	(4.6)	21	(2.6)	31	(9.7)	<0.001
**Primary diagnosis for HT**							<0.001
CHD	73	(6.5)	62	(7.7)	11	(3.4)	
Cardiomyopathy	452	(40.0)	291	(36.0)	161	(50.2)	
CAD	278	(24.6)	142	(17.6)	136	(42.4)	
Others	326	(28.9)	313	(38.7)	13	(4.0)	
Statin	305	(27.0)	196	(24.3)	109	(34.0)	<0.001
**Immunosuppressants** **Calcineurin inhibitors**							<0.001
No	393	(34.8)	371	(45.9)	22	(6.9)	
Cyclosporin	618	(54.7)	358	(44.3)	260	(81.0)	
Tacrolimus	118	(10.5)	79	(9.8)	39	(12.2)	
**mTOR inhibitors**							0.003
No	767	(67.9)	570	(70.5)	197	(61.4)	
Rapamycin	362	(32.1)	238	(29.5)	124	(38.6)	
**Antimetabolites**							<0.001
No	454	(40.2)	415	(51.4)	39	(12.2)	
MMF	484	(42.9)	328	(40.6)	156	(48.6)	
Azathioprine	191	(16.9)	65	(8.0)	126	(39.3)	

^a^: Chi-square test. Values expressed as number (percent) unless specified. Abbreviations: HT, heart transplantation; SD, standard deviation; HBV, hepatitis B virus; HCV, hepatitis C virus; DM, diabetes mellitus; CKD, chronic kidney disease; AKI, acute kidney injury; CHD, congenital heart disease; CAD, coronary artery disease; mTOR, mammalian target of rapamycin; MMF, Mycophenolate mofetil.

**Table 2:** Multivariate analysis of mortality between the dialysis and non-dialysis groups.

	Nondialysis Cohort				Dialysis Cohort				
Variables	Events	FT ^†^	Rate	Events	FT	Rate	HR ^‖^	(95% CI)	*p*
Outcome = Mortality									
Overall	311	4253	7.31	220	1361	16.16	3.44	(2.73–4.33)	<0.001
1y	111	747	14.86	116	235	49.36	5.89	(4.09–4.48)	<0.001
3y	208	1916	10.86	152	587	25.89	4.03	(3.03–5.34)	<0.001
5y	251	2773	9.05	170	852	19.95	3.68	(2.83–4.78)	<0.001

^†^ FT, follow-up time (years); Rate, per 100 person-years. ^‖^ HR, hazard ratio; adjusted for sex, age, and significant variables in Table 1.

**Table 3:** Multivariate analysis of mortality among the early, late, and non-dialysis groups.

						Overall			Dialysis Cohort		
Variables	*n*	(%)	Events	FT ^†^	Rate	HR ^‖^^‖^	(95% CI)	*p*	HR ^‖^^‖^	(95% CI)	*p*
Outcome = Mortality											
Dialysis timing ^††^											
Nondialysis	808	(71.6)	311	4253	7.31	1.00	(reference)				
Early	237	(21.0)	151	972	15.53	3.58	(2.74–4.67)	<0.001	1.00	(reference)	
Late	84	(7.4)	69	389	17.74	3.27	(2.44–4.36)	<0.001	1.23	(0.88–1.71)	0.220

^†^ FT, follow-up time (years); Rate, per 100 person-years. HR, hazard ratio; adjusted for sex, age, and significant variables in Table 1. ^††^ Dialysis timing: early dialysis denotes acute kidney injury requiring dialysis during the early postoperative period and before discharge; late dialysis denotes the need for dialysis after discharge. ^‖‖^ adjusted for sex, age, and significant variables in Table 1.

**Table 4:** Multivariate logistic regression analysis of risk factors associated with dialysis (non-dialysis vs. dialysis).

Variables	OR	(95% CI)	*p*-Value
**Demographics**			
Age (years)			
<18	1.49	(0.81–2.71)	0.203
18–39	0.80	(0.54–1.18)	0.264
40–59	1.00	(reference)	
≥60	1.17	(0.80–1.73)	0.418
Sex			
Female	1.00	(reference)	
Male	1.27	(0.89–1.81)	0.185
**Comorbidities (yes vs. no)**			
HBV	0.32	(0.14–0.76)	0.010
HCV	0.67	(0.21–2.21)	0.515
Cirrhosis	1.11	(0.76–1.63)	0.577
DM	1.50	(1.07–2.11)	0.019
CKD	2.65	(1.46–4.79)	0.001
Hypertension	0.95	(0.69–1.30)	0.746
Hyperlipidemia			
without statin	0.63	(0.38–1.07)	0.086
with statin	0.71	(0.47–1.06)	0.090
AKI	2.36	(1.27–4.41)	0.007
CHD	1.19	(0.68–2.07)	0.536
Cardiomyopathy	0.76	(0.53–1.10)	0.144
CAD	10.78	(6.33–18.4)	<0.001

OR, odds ratio, adjusted for all variables in Table 1. Abbreviations: HBV, hepatitis B virus; HCV, hepatitis C virus; DM, diabetes mellitus; CKD, chronic kidney disease; AKI, acute kidney injury; CHD, congenital heart disease; CAD, coronary artery disease.

**Table 5:** Multivariate logistic regression analysis of risk factors associated with early dialysis (non-dialysis vs. early dialysis).

Variables	OR	(95% CI)	*p*-Value
**Demographics**			
Age (years)			
<18	1.45	(0.73–2.90)	0.293
18–39	0.88	(0.58–1.35)	0.564
40–59	1.00	(reference)	
≥60	1.01	(0.64–1.58)	0.978
Sex			
Female	1.00	(reference)	
Male	1.39	(0.93–2.08)	0.112
**Comorbidities (yes vs. no)**			
HBV	0.37	(0.15–0.93)	0.034
HCV	0.72	(0.19–2.74)	0.632
Cirrhosis	1.20	(0.79–1.81)	0.402
DM	1.33	(0.91–1.96)	0.142
CKD	2.84	(1.54–5.24)	<0.001
HTN	0.79	(0.56–1.13)	0.203
Hyperlipidemia			
without statin	0.65	(0.36–1.18)	0.157
with statin	0.63	(0.40–0.99)	0.045
AKI	3.00	(1.56–5.78)	0.001
CHD	1.31	(0.71–2.42)	0.383
Cardiomyopathy	0.56	(0.38–0.83)	0.004
CAD	17.29	(9.17–32.6)	<0.001

OR, odds ratio, adjusted for all variables in Table 1. Abbreviations: HBV, hepatitis B virus; HCV, hepatitis C virus; DM, diabetes mellitus; CKD, chronic kidney disease; AKI, acute kidney injury; CHD, congenital heart disease; CAD, coronary artery disease.

**Table 6:** Multivariate logistic regression analysis of risk factors associated with late dialysis (non-dialysis vs. late dialysis).

Variables	OR	(95% CI)	*p*-Value
**Demographics**			
Age (years)			
<18	0.78	(0.25–2.43)	0.674
18–39	0.51	(0.22–1.18)	0.114
40–59	1.00	(reference)	
≥60	1.30	(0.65–2.56)	0.458
Sex			
Female	1.00	(reference)	
Male	0.64	(0.32–1.30)	0.216
**Comorbidities (yes vs. no)**			
HBV	1.51	(0.16–14.3)	0.720
HCV	0.71	(0.07–7.46)	0.773
Cirrhosis	0.64	(0.30–1.37)	0.250
DM	1.17	(0.62–2.23)	0.625
CKD	0.58	(0.20–1.64)	0.300
HTN	1.69	(0.91–3.13)	0.096
Hyperlipidemia			
without statin	0.82	(0.29–2.34)	0.714
with statin	1.42	(0.67–3.00)	0.362
AKI	0.62	(0.20–1.92)	0.406
**Maintenance immunosuppressive drugs** **Calcineurin inhibitors**			
Cyclosporin	1.00	(reference)	
Tacrolimus	1.06	(0.38–2.95)	0.907
None	0.67	(0.21–2.18)	0.511
mTOR inhibitors			
Sirolimus (Rapamycin)	0.17	(0.08–0.36)	<0.001
Antimetabolites			
Azathioprine	1.00	(reference)	
MMF	0.26	(0.13–0.51)	<0.001
None	0.66	(0.25–1.76)	0.409

OR, odds ratio, adjusted for all variables in Table 1. Abbreviations: HBV, hepatitis B virus; HCV, hepatitis C virus; DM, diabetes mellitus; CKD, chronic kidney disease; AKI, acute kidney injury; MMF, Mycophenolate mofetil.

## Supplementary Materials

The following is available online at https://www.mdpi.com/2077-0383/9/8/2455/s1, Figure S1: Comparison of long-term survival according to primary diagnosis for heart transplantation, Table S1: Comparison of risk factors predicting early postoperative AKI between Taiwan National Cohort and published studies.
