# Supplementary material for: Long-Term Outcomes and Risk Factors of Renal Failure Requiring Dialysis after Heart Transplantation: A Nationwide Cohort Study
Source: J Clin Med. 2020 Jul 31;9(8):2455. doi: 10.3390/jcm9082455 (PMC7464655; doi:10.3390/jcm9082455)
Supplement: Supplementary file 1 [file jcm-09-02455-s001.pdf]

Journal of
Clinical Medicine

MDPI

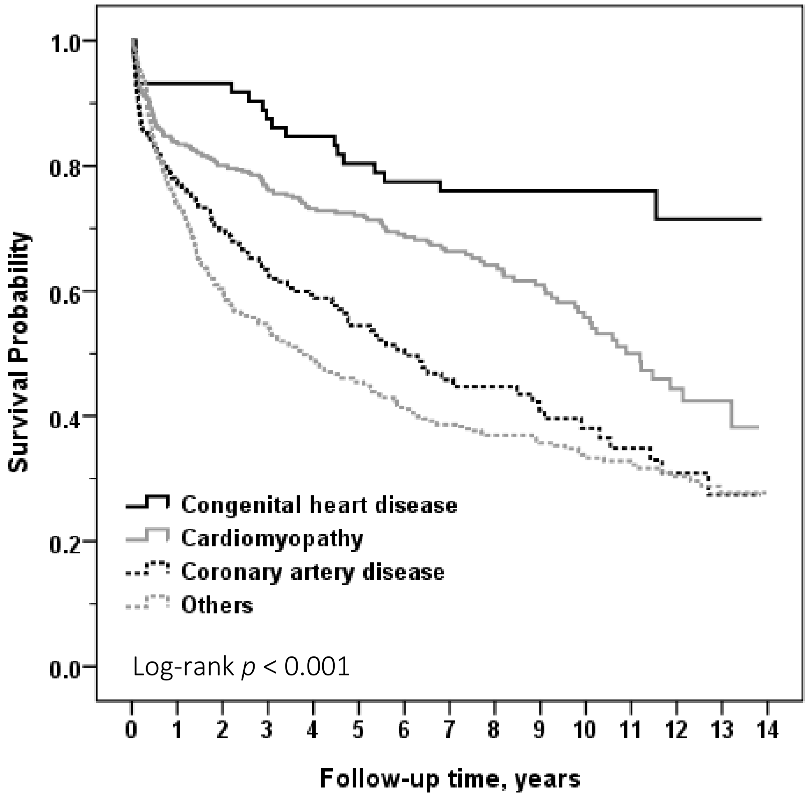

Figure S1. Comparison of long-term survival according to primary diagnosis for heart transplantation.

Table S1. Comparison of risk factors predicting early postoperative AKI between Taiwan National Cohort and published studies.

Author (patient number)	Wang (*n* = 1129)	Jokinen[14] (*n* = 93)	Boyle[18] (*n* = 756)	De Santo[42] (*n* = 307)	García-Gigorro[23] (*n* = 310)	Gude[24] (*n* = 585)	Fortrie[12] (*n* = 531)
Year/Country	2020/Taiwan	2010/Finland	2006/USA	2011/Italy	2018/Spain	2010/Norway	2016/Netherlands
Research setting	National cohort; retrospective	Single center; retrospective	Single center; retrospective	Single center; retrospective	Single center; retrospective	Single center; retrospective	Single center; retrospective
Age in years	46 ± 17*	49 ± 12*	57 (50, 63)**	32 ± 12*	53**	50 ± 12*	51**
Male	70%	70%	80%	67%	80%	NA	78%
Follow-up in years	5.0 ± 4.1*	Up to 1 year	Hospital stay	Hospital stay	6.7**	6.6 ± 5.5*	Up to 1 year
Definition of AKI	AKI-D	AKI-D	AKI-D	RIFLE-I/F (> 50% decrease in eGFR or AKI-D)	KDIGO criteria: AKI stage 1-3	AKIN (> 50% increase in sCr or AKI-D)	KDIGO criteria: AKI stage 1-3
AKI incidence	21%	25%	6%	14%	40% (AKI-D: 10.3%)	25% (AKI-D: 12%)	76% (AKI-D: 5%)
Risk factors: pre-operative	CKD<br>AKI<br>CAD	ARB use<br>Pacemaker<br>Time $_{\text{Intubation}}$ >24 hours<br>eGFR < 60 ml/min/1.73 m^2	High sCr<br>Insulin-requiring DM	Previous heart operation	Logistic EuroSCORE	Pre-operative cardiac output	BMI<br>DM<br>CKD stage by eGFR
Risk factors: Peri- or post-operative	NA	PGD<br>Re-admission to OR<br>Diuresis during surgery<br>Post-operative increase troponin T	Cardiopulmonary bypass time	Blood transfusion > 4 units<br>Elevated Troponin I<br>Length of ischemic time	Cardiac tamponade<br>Acute RV failure Major bleeding	Donor age<br>IV CsA immediately post-operatively use	RV failure
Protective factors	HBV<br>Statin use<br>Cardiomyopathy		Higher serum albumin				Higher age,<br>Induction therapy
Follow-up in years	5.0 ± 4.1*	Up to 1 year	Hospital stay	Hospital stay	6.7**	6.6 ± 5.5*	Up to 1 year
Short-term/intermediate survival impact of AKI‖	In hospital ↓	NA	In hospital ↓	In hospital ↓<br>1-year ↓	In hospital ↓	3-month ↓	1-year ↓ (only in AKI-D)
Long-term survival impact of AKI	NS for those who survived 3 months	NA	NA	NA	NS††	NS for those who survived 3 months	NA

*Values expressed as mean ± standard deviation; **Values expressed as median (25th percentile, 75th percentile if data are available); ‖Compare with non-AKI cohort unless otherwise stated; ††Survival after discharge compared with patients with AKI but no RRT requirement. Abbreviations: AKI, acute kidney injury; AKI-D, acute kidney injury requiring dialysis; NA, not available;

RIFLE-I/F, RIFLE (Risk, Injury, Failure, Loss, and End-stage kidney disease) criteria-Injury or Failure; eGFR, estimated glomerular filtration rate; KDIGO, Kidney Disease: Improving Global Outcomes; AKIN, the Acute Kidney Injury Network; sCr, serum creatinine; CKD, chronic kidney disease; CAD, coronary artery disease; ARB, angiotensin receptor blocker; DM, diabetes mellitus; EuroSCORE, European System of Cardiac Operative Risk Evaluation; BMI, body mass index; PGD, Prolonged graft dysfunction; OR, operating room; RV, right ventricular; IV CsA, intravenous cyclosporine; HBV, hepatitis B virus; HCV, hepatitis C virus; AKI, acute kidney injury; MMF, Mycophenolate mofetil; NS, nonsignificant.
